# Frailty elements, direct oral anticoagulants and mortality risk in atrial fibrillation patients aged ≥ 80 or ≥ 90 years from the nationwide Italian START registry: a propensity score matching analysis

**DOI:** 10.1007/s11357-025-01936-8

**Published:** 2025-10-22

**Authors:** Danilo Menichelli, Emilia Antonucci, Daniela Poli, Francesco Baratta, Angela Sciacqua, Giuseppe Armentaro, Gianluca Gazzaniga, Federica Moscucci, Gualtiero Palareti, Alessio Farcomeni, Evaristo Ettorre, Giovambattista Desideri, Francesco Violi, Pasquale Pignatelli, Daniele Pastori, START2 Register Investigators

**Affiliations:** 1 Department of General Surgery, Surgical Specialty and Anesthesiology, Sapienza University of Rome, Rome, Italy; 2 Arianna Anticoagulazione Foundation, Bologna, Italy; 3 Department of Critical Care Medicine, Thrombosis Centre, Azienda Ospedaliera Universitaria Careggi, Florence, Italy; 4 Geriatric Unit, Department of Internal Medicine and Medical Specialties, AOU Policlinico Umberto I, 00161 Rome, Italy; 5 Geriatrics Division, “Renato Dulbecco” University Hospital of Catanzaro, 88100 Catanzaro, Italy; 6 Department of Medical and Surgical Sciences, University Magna Græcia of Catanzaro, Catanzaro, Italy; 7 Department of Economics and Finance, University of Rome “Tor Vergata,”, Rome, Italy; 8 Department of Medical and Cardiovascular Sciences, Sapienza University of Rome, Rome, Italy; 9 IRCCS Neuromed, Località Camerelle, Pozzilli, Isernia Italy

**Keywords:** Anticoagulants, Atrial fibrillation, Cardiovascular events, Mortality

## Abstract

This study aims to investigate the use of direct oral anticoagulants (DOACs) in elderly frail patients with atrial fibrillation (AF). In 4244 patients aged ≥ 80 years and 533 aged ≥ 90 years from the nationwide START registry, we examined factors associated with DOACs use (vs warfarin) and its association with all-cause and cardiovascular (CV) death, CV events (CVEs) and bleeding. Propensity score-matching (PSM) analysis was also performed. Mean age was 84.8 years, 54.9% were women and 64% received DOACs. Frailty elements as tendency to falls (odds ratio [OR] 0.66, *p* = 0.030), living alone (OR 0.74, *p* = 0.027) and lack of familial/social support (OR 0.74, *p* < 0.001) were inversely associated with DOACs use, while wheelchair use was directly associated (OR 1.57, *p* = 0.014). During a mean of 686 ± 538 days follow-up, 492 all-cause deaths, 110 CV deaths, 548 CVEs and 221 bleedings occurred. Multivariable Cox regression and Fine–Gray analyses showed that DOAC use inversely associated with all-cause mortality (hazard ratio [HR] 0.35, *p* < 0.001), CV death (subdistribution HR [sHR] 0.43, *p* < 0.001), CVEs (sHR 0.46, *p* < 0.001) but not with bleeding. These associations were confirmed after PSM and persisted between sexes, in patients with Charlson Comorbidities Index > 5 or aged ≥ 90 years. At 24 months, the number needed to treat (NNT) for all-cause, CV death and CVEs were 11.0, 40.2 and 13.4. In patients aged ≥ 90 years, NNTs were 4.2, 4.5 and 12.0. In very elderly AF patients, DOACs prescription is influenced by frailty elements. DOACs inversely associated with mortality and CVEs risk independently from age, sex and comorbidities burden.

**Graphical Abstract:**

**Figure:**
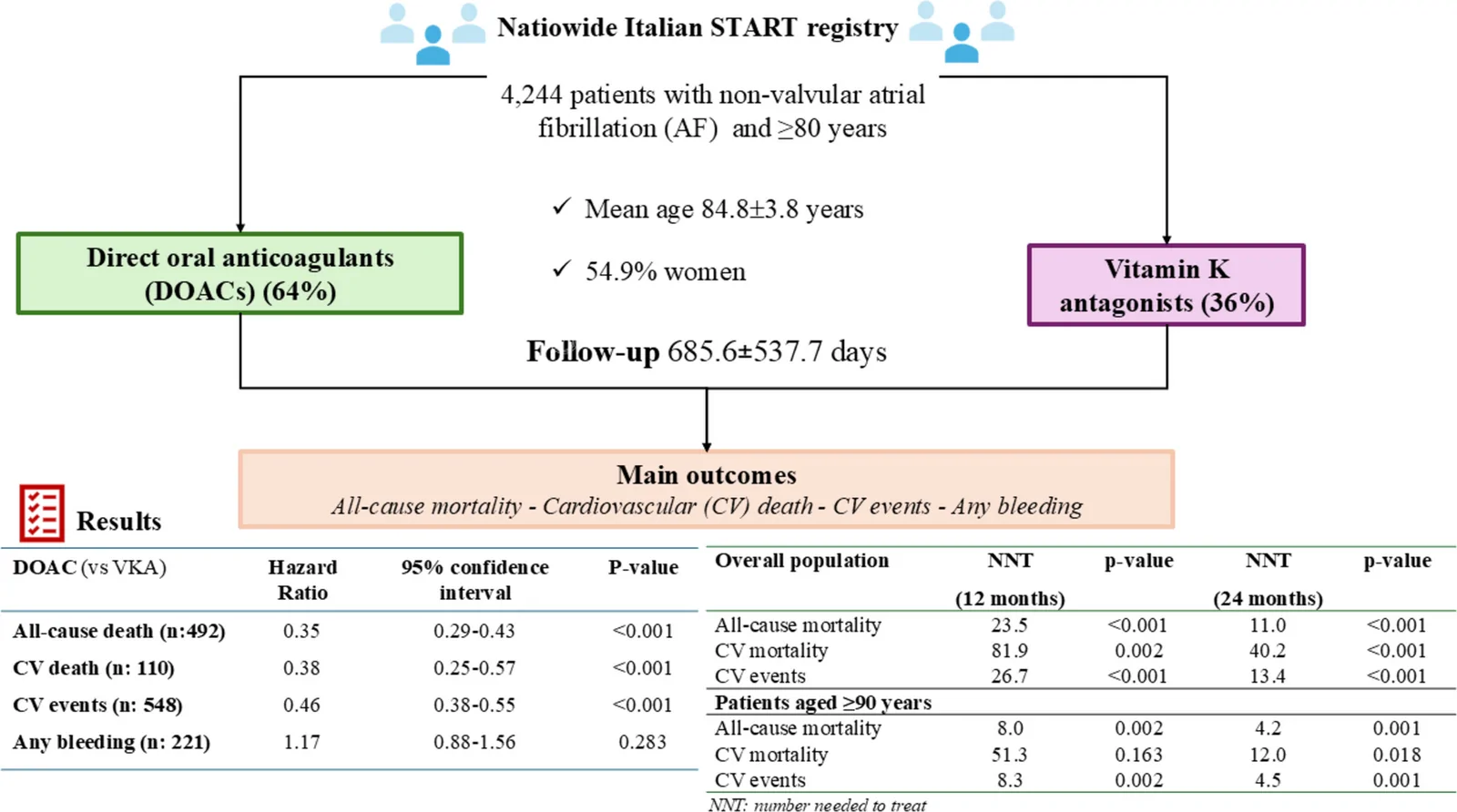

**Supplementary Information:**

The online version contains supplementary material available at https://doi.org/10.1007/s11357-025-01936-8.

## Introduction

Epidemiological and clinical studies consistently demonstrated that atrial fibrillation (AF) is an age-dependent disease[1]. Indeed, the prevalence of AF raises from 1 to 2% in the general population to > 10% in patients aged 80 years or elderly both in Western[2] and Eastern countries[3]. The risk of thromboembolic ischemic stroke also increases by age[4]. Indeed, even in absence of other non-sex related risk factors the risk of ischemic stroke increases from 0.7% to 1.7% at 1 year in AF patients in the absence of anticoagulation[5].

The role of oral anticoagulation in preventing AF-related thromboembolism is well established, with direct oral anticoagulants (DOACs) representing the best choice in most patients, with a similar benefit compared to vitamin K antagonists (VKAs) and lower risk of major bleeding, especially intracranial haemorrhages[6]. For this reason, in absence of contraindication (such as mechanical valve or mitral stenosis), latest European Society of Cardiology (ESC) guidelines recommend prescribing DOACs as first choice oral anticoagulants[7].

The management of oral anticoagulation in elderly patients (i.e. patients aged 80 or 90 years old) with AF is less clear. The use of oral anticoagulant in this high-risk population is more challenging as there is a concern for the increased risk of bleeding. Indeed, the risk of major bleeding increases from 1.8% to 4.8% in patients treated with warfarin[8], especially when geriatric conditions, such as frailty, history of falls and depression, coexist[9–11]. For some of these elements, limited evidence on DOACs exist[12].

Phase 3 randomized trials of oral anticoagulants included a proportion of elderly patients ranging from 31 to 43% (defined by age ≥ 75 years) not providing solid evidence on the management of patients over 80 or 90 years[13].

For these reasons, many elderly patients are left untreated or are given antiplatelet agents[14] instead of anticoagulants because of a perceived lower risk of bleeding[15, 16]. As a matter of fact, some cohort studies showed a proportion of elderly AF patients left untreated > 50%[17]. This therapeutic approach translates into an increased risk of both ischemic and bleeding events[16]. Regarding oral anticoagulation treatment, it is unclear which factors affect the choice of DOACs over VKAs in this high-risk population in routine clinical practice. This is even more important considering the recently published results from the randomized FRAIL-AF trial, which showed that switching from VKAs to DOAC in frail AF patients was associated with an increased risk of bleeding without a reduction in thromboembolism[18].

We investigated this aspect using data from the large, nationwide START registry, which includes patients with non-valvular AF starting oral anticoagulants. Our analysis focused on identifying factors associated with the use of DOACs over VKAs in patients aged 80 years or elderly.

## Methods

### START registry

The START registry is an observational, prospective, multicentre, ongoing cohort study that includes patients (aged ≥ 18 years) who start anticoagulation therapy, either VKAs or DOACs, throughout Italy. Data analysed in this paper are of patients enrolled until December 2023. Details of the START registry have been previously described[19].

Only AF patients ≥ 80 years were included in the analysis. Patients treated with low-molecular-weight heparin were excluded as well as patients already enrolled in phase 2 or 3 clinical studies. Patients enrolled in other observational, or phase 4 studies, were considered eligible for the study.

### Baseline characteristics

Patient’s clinical features are recorded by participants on web-based case report forms (CRF). Baseline data are demographic and clinical characteristics of patients, including cardiovascular risk factors, comorbidities such as peripheral artery disease (PAD), heart failure (HF), chronic kidney disease (CKD) and coronary artery disease (CAD), laboratory routine data, smoking habits, indication for anticoagulant treatment, type of oral anticoagulation and concomitant drugs. CAD was defined as history of coronary artery disease (either ischemic heart disease or coronary revascularization with stent or coronary artery bypass graft), while cerebrovascular disease is defined as previous ischemic stroke or transient ischaemic attack (TIA). Heart failure (HF) was defined as: Medical history of hospital admission for HF and/or symptoms and signs suggestive for HF. CKD was defined according to an estimated glomerular filtration rate (eGFR) < 60 ml/min. Peripheral artery disease was defined according to ESC guidelines[20].

### Study endpoints

The endpoints of the study were (1) to investigate factors associated with the choice of DOACs (vs VKAs), (2) to evaluate the association between DOAC use with adverse clinical events such as all-cause and cardiovascular (CV) mortality, CVEs (defined as fatal/non-fatal myocardial infarction, cardiac revascularization, fatal or non-fatal ischemic stroke and transient ischemic attack) and any bleeding in the whole cohort and in the subgroup of patients aged ≥ 90 years.

### Ethics statement

The START registry has been previously registered on ClinicalTrials.gov (NCT02219984). The study protocol was accepted by the Institutional Review Board of each participating centre, and informed consent was obtained from patients at enrollment. The study protocol complies with the ethical guidelines of the 1975 Helsinki Declaration, and informed consent was obtained from each patient.

### Patient and public involvement statement

Patients and members of the public were not involved in the design, conduct, reporting or dissemination plans of this research. They did not contribute to the development of the research question, outcome measures or study design, nor were they involved in participant recruitment or in assessing the burden of the intervention.

### Statistical analysis

Continuous variables were reported as mean and standard deviation. Group comparisons were performed by unpaired Student’s *t* test or analysis of variance (ANOVA). Proportions and categorical variables were compared using the chi-squared test.

Population characteristics have been described according to the use of DOACs or VKAs. A multivariable logistic regression to establish clinical factors associated with DOAC use was performed including all variables evaluated at baseline. Results of the multivariable logistic regression were expressed as odds ratio (OR) and 95% confidence interval (95%CI).

The cumulative incidence of adverse clinical events was estimated using a Kaplan–Meier product-limit estimator. Survival curves were formally compared using the log-rank test.

Univariable and multivariable stepwise Cox proportional hazards regression analyses were conducted to estimate adjusted hazard ratios (HRs) and 95% CIs for all-cause mortality associated with each clinical variable. To account for competing risks, both univariable and multivariable Fine–Gray subdistribution hazard models were employed to estimate the cumulative incidence of other cardiovascular outcomes. Specifically, non-cardiovascular (non-CV) death was treated as a competing event for CV mortality, while all-cause mortality was considered a competing event for CVEs and bleeding outcomes. Results from competing risk analyses were reported as subdistribution hazard ratios (sHRs) with corresponding 95% CIs.

The number needed to treat (NNT) for all-cause of death, CV death and CVEs and their 95%CI were calculated at 12 and 24 months of follow-up for the overall population and in patients aged 90 years or elderly.

To address baseline differences between patients treated with VKAs and DOACs, we also performed a propensity score matching (PSM) analysis, with the aim of assessing consistency of results across different modelling strategies. The propensity score was estimated using logistic regression, incorporating the following covariates: age, sex, paroxysmal AF, CAD, HF, PAD, CKD, alcohol use, dementia, use of a wheelchair, living alone and family support. Patients were matched 1:1 using nearest-neighbour matching without replacement, applying a caliper of 0.1. Covariate balance after matching was assessed using standardized mean differences (SMDs), with values < 0.15 considered indicative of adequate balance. Balance diagnostics through a Love plot is shown in Supplementary Fig. 1.

Following matching, we evaluated overall mortality using a Cox proportional hazards model, while CV death, CVEs and major bleeding were analysed using Fine–Gray subdistribution hazard models, accounting for competing risks as previously described.

Subgroup analyses were conducted for patients aged ≥ 90 years, according to the specific type of DOAC used, sex, frailty (defined as the presence of at least one frailty elements among dementia, immobilization syndrome, wheelchair use, tendency to fall and living alone), Charlson Comorbidity Index (CCI)[21], with a clinically relevant threshold of 5 for higher risk[22].

Only *p* values < 0.05 were considered statistically significant. All tests were two-tailed, and analyses were performed using computer software packages (IBM SPSS-25, SPSS Inc., R software version 4.2.3, and MedCalc).

## Results

### Cohort characteristics

Overall, 10,369 patients were enrolled in the START registry up to December 2023. After excluding those < 80 years, 4244 patients (40.9%) were included in the analyses. The mean age was 84.8 ± 3.8 years and 54.9% of patients were women. Overall, 2716 (64%) were treated with DOACs. The distribution of DOACs was apixaban (34.9%), rivaroxaban (23.7%), edoxaban (22.6%) and then dabigatran (18.6%).

Compared to VKAs, patients on DOACs were more frequently affected by paroxysmal AF, dementia, immobilization syndrome, wheelchair users and had a higher social/familial support (Table 1). Moreover, they were treated with class 1c antiarrhythmics and antipsychotic drugs.

**Table 1:** Patients’ characteristics according to anticoagulant prescription

	Total cohort (*n*: 4244)	VKA (*n*: 1528)	DOAC (*n*: 2716)	*p* value
Age (years)	84.8 ± 3.8	84.1 ± 3.2	85.1 ± 4.0	< 0.001
Women (%)	2331 (54.9)	845 (55.3)	1486 (54.7)	0.712
Paroxysmal AF (%)	1300 (30.9)	407 (27.4)	893 (32.9)	< 0.001
Arterial hypertension (%)	3572 (84.2)	1270 (83.1)	2302 (84.8)	0.160
Diabetes (%)	814 (19.2)	287 (18.8)	527 (19.4)	0.622
Previous CAD (%)	720 (17.0)	316 (20.7)	404 (14.9)	< 0.001
Previous cerebrovascular disease (%)	736 (17.3)	257 (16.8)	479 (17.6)	0.500
Obesity* (%)	623 (14.7)	207 (13.5)	416 (15.3)	0.118
Anaemia (%)	1475 (34.8)	547 (35.8)	928 (34.2)	0.284
Heart failure (%)	1101 (25.9)	439 (28.7)	662 (24.4)	0.002
Cancer (%)	699 (16.5)	257 (16.8)	442 (16.3)	0.646
Peripheral artery disease (%)	264 (6.2)	119 (7.8)	145 (5.3)	0.002
COPD/OSAS (%)	543 (12.8)	200 (13.1)	343 (12.6)	0.667
CKD** (%)	1000 (23.6)	301 (19.7)	699 (25.8)	< 0.001
Alcohol use (%)	201 (4.7)	107 (7.0)	94 (3.5)	< 0.001
CHA_2_DS_2_-VASc (mean)	4.3 ± 1.2	4.4 ± 1.2	4.3 ± 1.2	0.127
HAS-BLED (mean)	2.3 ± 0.7	2.3 ± 0.8	2.2 ± 0.7	0.001
Charlson Comorbidity Index (mean) *Charlson Comorbidity Index* ≥ *3 (%)*	5.7 ± 1.5 4244 (100.0)	5.7 ± 1.5 1528 (100.0)	5.7 ± 1.6 2716 (100.0)	0.513
Frailty elements				
Dementia (%)	241 (5.7)	64 (4.2)	177 (6.5)	0.007
Immobilization syndrome (%)	60 (1.4)	13 (0.9)	47 (1.7)	< 0.001
Wheelchair users (%)	207 (4.9)	54 (3.5)	153 (5.6)	0.009
Tendency to fall (%)	148 (3.5)	65 (4.3)	83 (3.1)	0.122
Living alone (%)	260 (6.1)	117 (7.7)	143 (5.3)	0.008
Social/familial support (%)	3059 (72.1)	1058 (69.2)	2001 (73.7)	0.001
Therapy				
Type of DOAC (%) *Apixaban* *Rivaroxaban* *Edoxaban* *Dabigatran*	949 (22.4) 645 (15.2) 615 (14.5) 506 (11.9)	- - - -	949 (34.9) 645 (23.7) 615 (22.7) 507 (18.7)	
Time in therapeutic range (TiTR) median [IQR]^+^	-	67.0 [56.0–76.0]	-	-
Concomitant aspirin (%)	493 (11.6)	219 (14.3)	274 (10.1)	< 0.001
Class 1c antiarrhythmics (%)	207 (4.9)	53 (3.5)	154 (5.7)	0.002
Amiodarone (%)	464 (10.9)	178 (11.6)	286 (10.5)	0.262
Lipid lowering therapy (%)	1351 (31.8)	472 (30.9)	879 (32.4)	0.323
RAAS inhibitors (%)	2353 (55.4)	832 (54.5)	1521 (56.0)	0.329
Beta blockers (%)	1932 (45.5)	834 (54.6)	1098 (40.4)	< 0.001
Calcium channel blockers (%)	972 (22.9)	364 (23.8)	608 (22.4)	0.285
Diuretics (%)	2021 (47.6)	752 (49.2)	1269 (46.7)	0.119
Digoxin (%)	427 (10.1)	176 (11.5)	251 (9.2)	0.018
Proton pump inhibitors (%)	1602 (37.7)	628 (41.1)	974 (35.9)	0.001
Antipsychotic drugs (%)	439 (10.3)	123 (8.0)	316 (11.6)	0.001
Anxiolytic drugs (%)	463 (10.9)	168 (11.0)	295 (10.9)	0.961
Antiepileptic drugs (%)	103 (2.4)	38 (2.5)	65 (2.4)	0.982

*AF atrial fibrillation, CKD chronic kidney disease, COPD/OSAS chronic obstructive pulmonary disease/obstructive sleep apnoea syndrome, DOAC direct oral anticoagulants, IQR interquartile range, RAAS renin–angiotensin–aldosterone inhibitors, VKA vitamin K antagonist*

^*^Defined as body mass index ≥ *30 kg/m*^*2*^

^**^Defined as estimated glomerular filtration rate (eGFR) < *60 ml/min*

^+^*Calculated according to Rosendaal method*[43]

Conversely, patients on VKAs had a higher proportion of patients with CAD, HF, PAD, CKD and more frequently were alcohol users (Table 1). They were also more frequently treated with concomitant antiplatelet, beta blockers, digoxin and proton pump inhibitors (Table 1).

### Clinical determinants of anticoagulant choice

At multivariable logistic regression analysis, factors directly associated with the use of DOACs were increasing age (OR 1.10, 95%CI 1.07–1.12, *p* < 0.001), arterial hypertension (OR 1.30, 95%CI 1.07–1.59, *p* = 0.009), paroxysmal AF (OR 1.33, 95%CI 1.14–1.54, *p* < 0.001) and CKD (OR 1.66, 95%CI 1.39–1.97, *p* < 0.001) (Table 2). On the other hand, the concomitant presence of atherosclerotic disease such as CAD (OR 0.73, 95%CI 0.60–0.89, *p* = 0.002) or PAD (OR 0.73, 95%CI 0.55–0.96, *p* = 0.022) and the alcohol use (OR 0.59, 95%CI 0.43–0.81, *p* = 0.001) were inversely associated with DOAC use.

**Table 2:** Multivariable logistic regression analysis of clinical factors associated with direct oral anticoagulants (DOACs) use (vs vitamin K antagonist)

	Odds ratio	95% confidence interval Low High		*p* value
Age	1.10	1.07	1.12	< 0.001
Female sex	0.95	0.83	1.10	0.519
Paroxysmal AF (vs persistent/permanent AF)	1.33	1.14	1.54	< 0.001
Arterial hypertension	1.30	1.07	1.59	0.009
Diabetes	1.08	0.90	1.28	0.418
Previous CAD	0.73	0.60	0.89	0.002
Previous cerebrovascular disease	1.00	0.83	1.19	0.962
Obesity*	1.07	0.88	1.30	0.521
Anaemia	0.91	0.79	1.06	0.219
Heart failure	0.87	0.74	1.03	0.103
Cancer	0.97	0.81	1.16	0.719
Peripheral artery disease	0.73	0.55	0.96	0.022
COPD/OSAS	1.05	0.85	1.28	0.675
CKD**	1.66	1.39	1.97	< 0.001
Dementia	1.35	0.98	1.87	0.065
Alcohol use	0.59	0.43	0.81	0.001
Immobilization syndrome	1.36	0.69	2.67	0.377
Wheelchair users	1.57	1.10	2.25	0.014
Tendency to fall	0.66	0.45	0.96	0.030
Living alone	0.74	0.56	0.97	0.027
No social/familial support	0.74	0.64	0.87	< 0.001
Concomitant aspirin (%)	0.74	0.60	0.92	0.008
Class 1c antiarrhythmics (%)	1.56	1.11	2.21	0.011
Amiodarone (%)	0.89	0.72	1.11	0.296
Lipid lowering therapy (%)	1.40	1.19	1.65	< 0.001
RAAS inhibitors (%)	1.01	0.88	1.17	0.848
Beta blockers (%)	0.59	0.51	0.67	< 0.001
Calcium channel blockers (%)	0.84	0.72	0.99	0.035
Diuretics (%)	0.99	0.85	1.14	0.837
Digoxin (%)	1.00	0.79	1.25	0.969
Proton pump inhibitors (%)	0.82	0.71	0.95	0.007
Antipsychotic drugs (%)	1.39	1.10	1.76	0.006
Anxiolytic drugs (%)	0.94	0.75	1.17	0.560
Antiepileptic drugs (%)	0.88	0.57	1.36	0.566

*AF atrial fibrillation, CKD chronic kidney disease, COPD/OSAS chronic obstructive pulmonary disease/obstructive sleep apnoea syndrome, DOAC direct oral anticoagulants, RAAS renin–angiotensin–aldosterone inhibitors*

^*^Defined as body mass index ≥ *30 kg/m*^*2*^

^**^Defined as estimated glomerular filtration rate (eGFR) < *60 ml/min*

Of note, frailty elements such as tendency to falls (OR 0.66, 95%CI 0.45–0.96, *p* = 0.030), living alone (OR 0.74, 95%CI 0.56–0.97, *p* = 0.027) and the absence of familial/social support (OR 0.74, 95%CI 0.64–0.87, *p* < 0.001) were inversely associated with the use of DOACs. Conversely, patients with reduction of mobility such as wheelchair users were more treated with DOACs (OR 1.57, 95%CI 1.10–2.25, *p* = 0.014) (Table 2).

Among treatments, the use of antiplatelets (OR 0.74, 95%CI 0.60–0.92, *p* = 0.008), beta blockers (OR 0.59, 95%CI 0.51–0.67, *p* < 0.001), calcium channel blockers (0.84, 95%CI 0.72–0.99, *p* = 0.035) and proton pump inhibitors (OR 0.82, 95%CI 0.71–0.95, *p* = 0.007) was inversely associated to DOACs use. Otherwise, class 1c antiarrhythmics (OR 1.56, 95%CI 1.11–2.21, *p* = 0.011), lipid lowering therapy (OR 1.40, 95%CI 1.19–1.65, *p* < 0.001) and antipsychotic drugs (OR 1.39, 95%CI 1.10–1.76, *p* = 0.006) were associated to DOAC use (Table 2).

### Clinical outcomes

During a mean follow-up of 686 ± 538 days, there were 492 all-cause deaths, 110 cardiovascular (CV) deaths, 548 CV events (CVEs) and 221 bleeding events. In univariable Cox regression analysis, DOAC use was inversely associated with all-cause mortality (HR 0.40, 95%CI 0.33–0.48, *p* < 0.001) compared to VKA. Using Fine and Gray competing risk models, DOAC use was also associated with a lower subdistribution hazard of CV death (sHR 0.48, 95%CI 0.32–0.71, *p* < 0.001) and CVEs (sHR 0.50, 95%CI 0.42–0.60, *p* < 0.001). No significant difference was observed between DOACs and VKAs for bleeding events (sHR 1.21, 95%CI 0.92–1.60, *p* = 0.2).

**Fig. 1:**
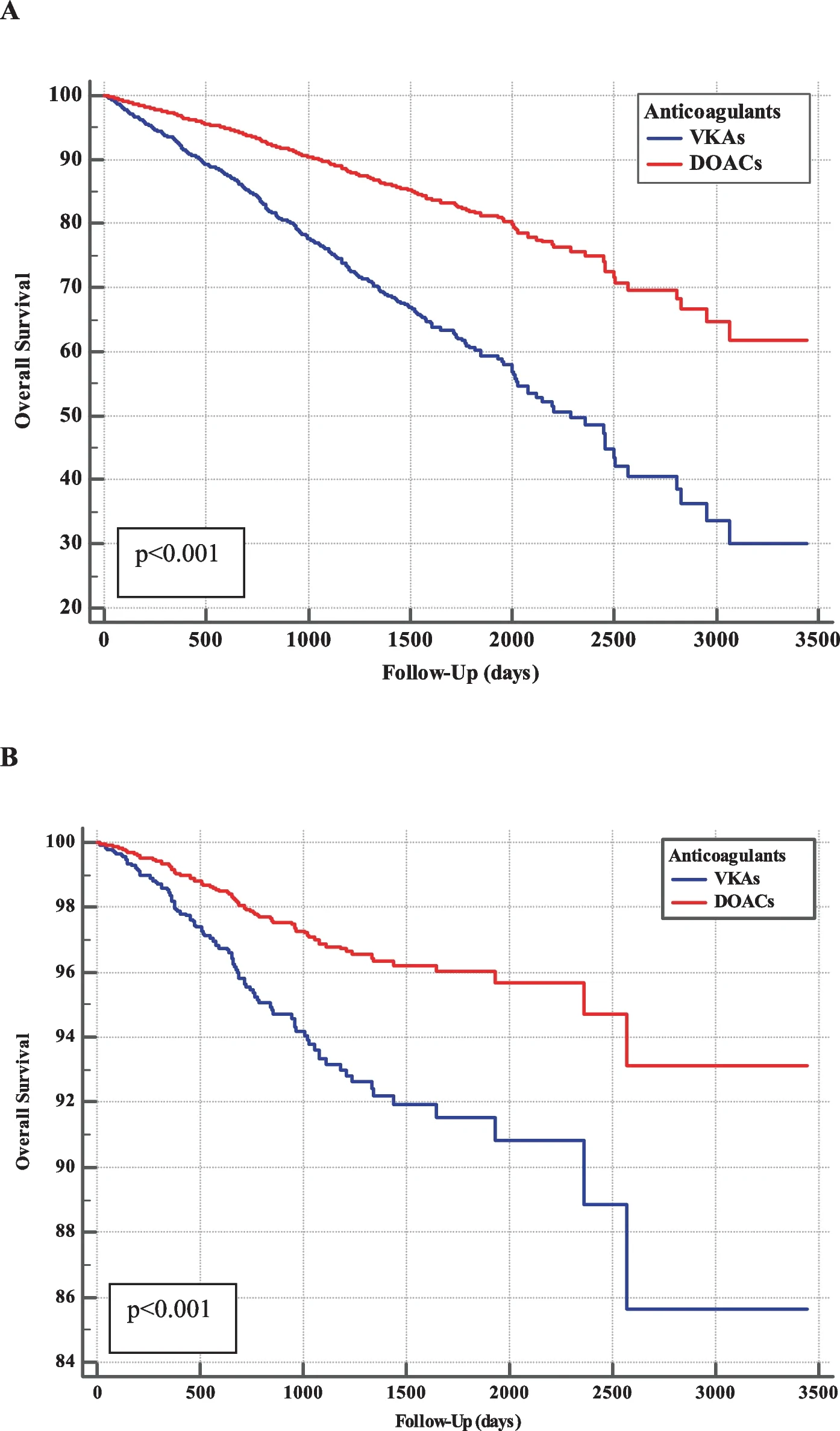

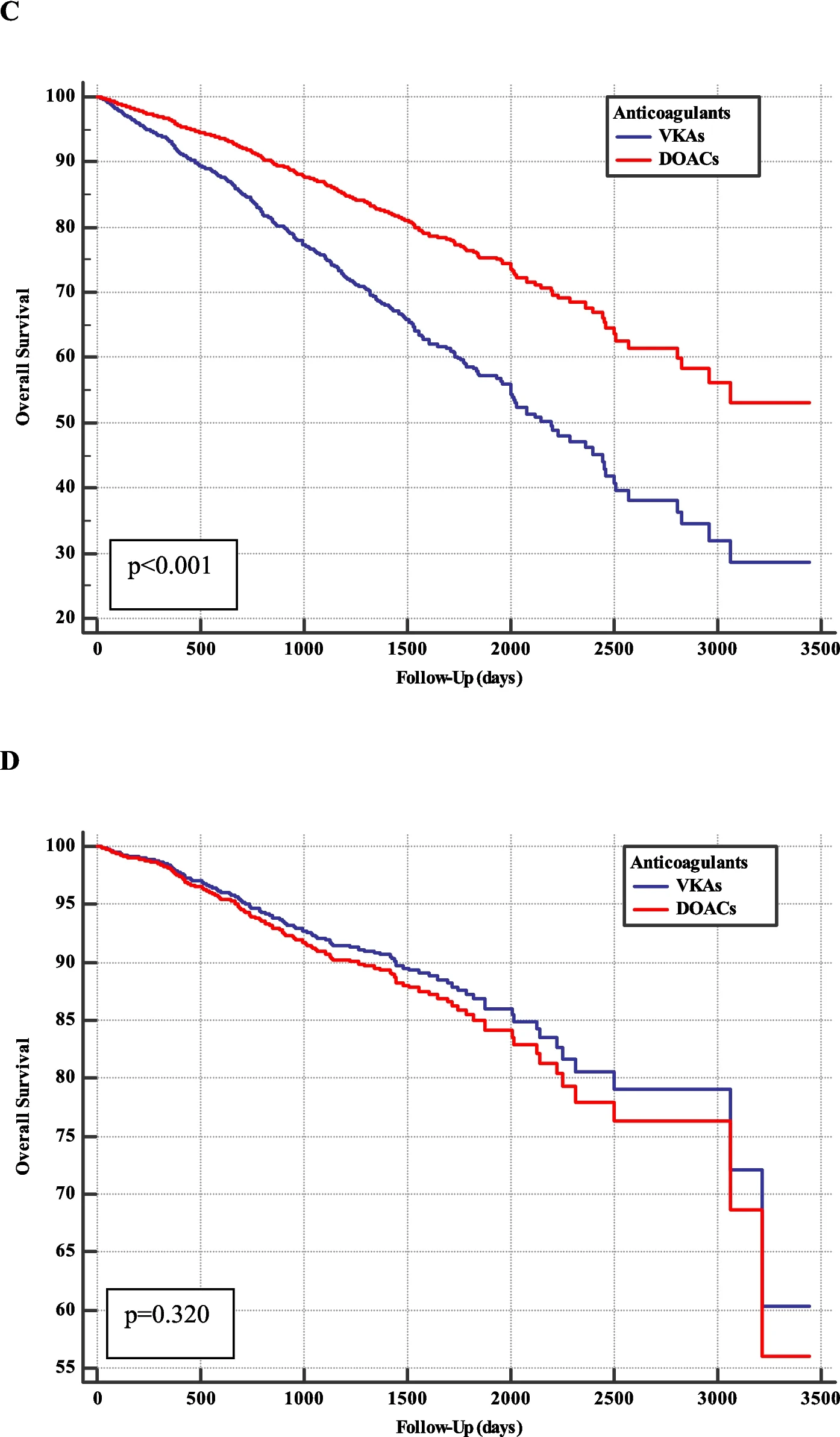
Kaplan–Meier curves for the type of anticoagulants for all-cause of mortality (Panel A), cardiovascular death (Panel B), cardiovascular events (Panel C) and any bleeding (Panel D). (*p* values refer to log-rank test)

Figure 1A–D shows the Kaplan–Meier curves for each endpoint (*p* values refer to log-rank test).

All-cause death. At multivariable stepwise Cox regression analysis (Table 3, Panel A), age, CAD, anaemia, COPD/OSAS, PAD, dementia and the use of wheelchair were associated with a higher risk of all-cause death, while, female sex, paroxysmal AF, CKD, lipid lowering therapy, the absence of familial/social support, RAAS inhibitors and particularly DOAC use (HR 0.35, 95%CI 0.29–0.43, *p* < 0.001) were associated with lower all-cause mortality risk. The NNT for all-cause death (Table 4, Panel A) was 23.5 at 12 months (*p* < 0.001) and 11.0 at 24 months (*p* < 0.001).

**Table 3:** Multivariable analysis of clinical factors associated with all-cause of death (Panel A), cardiovascular death (Panel B), cardiovascular events (Panel C) and any bleeding (Panel D)

Panel A. All-cause death**	Hazard ratio	95% confidence interval		*p* value
*Age*	1.12	1.09	1.14	< 0.001
*Female sex*	0.71	0.59	0.86	< 0.001
*Diabetes*	1.24	0.99	1.56	0.064
*CAD*	1.42	1.12	1.79	0.003
*Paroxysmal AF (vs persistent/permanent AF)*	0.78	0.63	0.97	0.023
*Anaemia*	1.30	1.08	1.57	0.006
CKD*	0.71	0.55	0.93	0.012
*Previous stroke/TIA*	1.24	0.99	1.55	0.063
*COPD/OSAS*	1.85	1.48	2.31	< 0.001
*PAD*	1.48	1.08	2.02	0.015
*Dementia*	1.53	1.09	2.13	0.013
*Wheelchair users*	1.75	1.25	2.45	0.001
*No familial/social support*	0.79	0.64	0.98	0.029
*Lipid lowering therapy*	0.69	0.55	0.86	0.001
*RAAS inhibitors*	0.80	0.66	0.96	0.016
*DOAC (vs VKA)*	0.35	0.29	0.43	< 0.001
Panel B. Cardiovascular death***	sHR	95% confidence interval		*p* value
*Age*	1.10	1.05	1.16	< 0.001
*Female sex*	0.58	0.39	0.86	0.006
CKD*	0.54	0.29	1.01	0.053
Heart failure	1.67	1.11	2.50	0.013
*PAD*	1.88	1.04	3.42	0.038
*Dementia*	1.91	0.96	3.80	0.064
*Wheelchair users*	1.83	0.92	3.67	0.087
*Lipid lowering therapy*	0.65	0.42	1.02	0.060
*DOAC (vs VKA)*	0.43	0.28	0.66	< 0.001
Panel C. Cardiovascular events***	sHR	95% confidence interval		*p* value
*Age*	1.10	1.07	1.12	< 0.001
*Female sex*	0.68	0.57	0.82	< 0.001
*Diabetes*	1.24	1.00	1.53	0.052
*CAD*	1.34	1.07	1.68	0.012
*Paroxysmal AF (vs persistent/permanent AF)*	0.84	0.69	1.02	0.084
*Anaemia*	1.27	1.06	1.52	0.010
CKD*	0.64	0.50	0.82	< 0.001
*Previous stroke/TIA*	1.30	1.06	1.60	0.013
Heart failure	1.21	0.99	1.48	0.065
*COPD/OSAS*	1.71	1.37	2.15	< 0.001
*PAD*	1.57	1.17	2.12	0.003
*Dementia*	1.44	1.02	2.03	0.039
*Wheelchair users*	1.70	1.23	2.36	0.002
*No familial/social support*	0.86	0.58	1.26	0.400
*Lipid lowering therapy*	0.70	0.57	0.87	0.001
*RAAS inhibitors*	0.80	0.68	0.96	0.015
*DOAC (vs VKA)*	0.46	0.38	0.55	< 0.001
Panel D. Any bleeding***	sHR	95% confidence interval		*p* value
*Age*	1.02	0.99	1.06	0.200
*Female sex*	0.64	0.49	0.84	0.001
*CAD*	1.32	0.95	1.84	0.100
*Paroxysmal AF (vs persistent/permanent AF)*	1.36	1.03	1.81	0.031
*Anaemia*	1.36	1.04	1.78	0.025
*No familial/social support*	1.65	1.01	2.71	0.047
*RAAS inhibitors*	0.84	0.64	1.10	0.200
*DOAC (vs VKA)*	1.25	0.95	1.65	0.110

*AF atrial fibrillation, CAD coronary artery disease, CKD chronic kidney disease, COPD/OSAS chronic obstructive pulmonary disease/obstructive sleep apnoea syndrome, DOAC direct oral anticoagulants, PAD peripheral artery disease, RAAS renin–angiotensin–aldosterone inhibitors, sHR subdistribution Hazard Ratio, TIA transient ischemic attack, VKA vitamin K antagonist*

^*^Defined as estimated glomerular filtration rate (eGFR) < *60 ml/min*

^**^Multivariable Cox proportional hazard regression analysis

^***^Fine–Gray sub-distribution hazard models

**Table 4:** Number needed to treat (NNT) for all-cause mortality, cardiovascular death and cardiovascular events in overall population (Panel A) and in a subgroup of ≥ 90 years patients at 12 and 24 months of follow-up

A. Overall population	NNT (12 months)	*p* value	NNT (24 months)	*p* value
All-cause mortality	23.5	< 0.001	11.0	< 0.001
CV mortality	81.9	0.002	40.2	< 0.001
CV events	26.7	< 0.001	13.4	< 0.001
B. Patients aged ≥ 90 years				
All-cause mortality	8.0	0.002	4.2	0.001
CV mortality	51.3	0.163	12.0	0.018
CV events	8.3	0.002	4.5	0.001

*CI confidence interval, CV cardiovascular*

CV death. In the multivariable Fine–Gray model, which accounted for non-cardiovascular death as a competing event, age, HF and PAD were independently associated with a higher risk of CV death (Table 3, Panel B); in contrast, female sex and DOAC use (sHR 0.43, 95%CI 0.28–0.66, *p* < 0.001) were associated with a lower risk. The NNT for CV death (Table 4, Panel A) at 12 months was 81.9 (*p* = 0.002) and at 24 months was 40.2 (*p* < 0.001).

CVEs. In the multivariable Fine–Gray model, accounting for all-cause mortality as a competing event, age, diabetes, CAD, anaemia, COPD/OSAS, PAD, dementia, previous stroke/TIA and the use of wheelchair were associated with a higher risk of CVEs (Table 3, Panel C); conversely, female sex, CKD, lipid lowering therapy, the absence of familial/social support, use of RAAS inhibitors and DOAC therapy (sHR 0.46, 95%CI 0.38–0.55, *p* < 0.001) were associated with lower risk. The NNT for CVEs (Table 4, Panel A) at 12 and 24 months was 26.7 (*p* < 0.001) and 13.4 (*p* < 0.001).

Bleeding. In multivariable Fine–Gray model, accounting for all-cause mortality as a competing event, paroxysmal AF, anaemia and no familial/social support were associated with an increased risk of bleeding events, while female sex was inversely associated (Table 3, Panel D). Of note, DOAC use was not significantly associated with bleeding compared to VKAs (sHR 1.25, 95%CI 0.95–1.65, *p* = 0.110).

PSM analysis.

Summary statistics for matched and unmatched cohorts are shown in Supplementary Table 1. After PSM, 1496 patients treated with VKAs and 1496 patients treated with DOACs were included, with all baseline characteristics achieving adequate balance based on SMDs. In the Cox proportional hazards model applied to the matched cohort, treatment with DOACs remained significantly associated with a lower risk of all-cause mortality (HR 0.37, 95%CI 0.29–0.47, *p* < 0.001). Similarly, in Fine–Gray competing risks models, DOAC use was associated with a reduced sHR for CV death (sHR 0.40, 95%CI 0.24–0.66, *p* < 0.001) and CVEs (sHR 0.50, 95%CI 0.40–0.62, *p* < 0.001). No significant difference was observed in the risk of major bleeding (sHR 1.26, 95% CI 0.91–1.74, *p* = 0.200).

### *Patients aged* ≥ *90 years*

In our cohort, 533 AF patients were aged ≥ 90 years, of whom 428 (80.3%) were on DOACs. During follow-up, there were 82 all-cause of deaths, 19 CV deaths, 89 CVEs and 28 bleeding events.

Kaplan–Meier curves for these outcomes are available in Supplementary Fig. 2, Panel A–D.

At univariable Cox regression analysis, DOAC use was associated with a significantly lower risk of all-cause mortality (HR 0.25, 95%CI 0.16–0.39, *p* < 0.001). In Fine–Gray models accounting for prespecified competing risks, DOAC use was also associated with reduced risk of CV death (sHR 0.18, 95% CI 0.07–0.42; *p* < 0.001) and CVEs (sHR 0.30, 95% CI 0.20–0.45; *p* < 0.001), compared to VKA use. No significant difference was observed for bleeding events (sHR 1.65, 95% CI 0.59–4.66; *p* = 0.340).

We evaluated also the NNT in this subgroup of very elderly patients and we found a NNT (Table 4, Panel B) for all cause of death and CVEs at 12 months of 8.0 (*p* = 0.002) and 8.3 (*p* = 0.002), respectively, and a NNT at 24 months of 4.2 (*p* = 0.001) and 4.5 (*p* = 0.001), respectively, while CV death (Table 4, Panel B) had a favourable NNT only at 24 months (NNT 12.0, *p* = 0.018).

### Type of DOAC

We found that all DOACs were associated with a lower risk of all-cause mortality (Supplementary Table 2, Panel A) and CVEs (Supplementary Table 2, Panel C), but only apixaban and edoxaban were also associated with a lower risk of CV deaths (Supplementary Table 2, Panel B). Of note, all DOACs seem to be not associated with a lower risk of bleeding events compared to VKAs (Supplementary Table 2, Panel D). A comparison between Xa and the IIa inhibitor dabigatran showed no relevant differences for the association with all-cause mortality (Supplementary Table 3, Panel A), CVEs (Supplementary Table 3, Panel C) and bleeding events (Supplementary Table 3, Panel D). Only edoxaban was associated with a slight reduction of CV deaths (sHR 0.21, 95%CI 0.05–0.99, *p* = 0.048) (Supplementary Table 3, Panel B). The results of subgroup analysis were coherent after adjustment for CHA_2_DS_2_-VASc.

### Sex

At Kaplan–Meier curves, a lower incidence of all-cause of death (Log-Rank test *p* < 0.001 for both subgroups), CV death (Log-Rank test *p* = 0.001 and *p* = 0.021, respectively) and CVEs (Log-Rank test *p* < 0.001 for both subgroups) was observed in both women and men treated with DOACs, while no difference was observed about bleeding events (Log-Rank test *p* = 0.518 and *p* = 0.487, respectively).

At univariable Cox regression analysis, DOAC use was associated with a significantly lower risk of all-cause mortality (HR 0.46, 95%CI 0.35–0.60, *p* < 0.001 and HR 0.34, 95%CI 0.26–0.45, *p* < 0.001, in both men and women respectively). At Fine–Gray univariable analysis, DOAC use was inversely associated with CV death (sHR 0.57, 95%CI 0.33–0.97, *p* = 0.039 and sHR 0.38, 95%CI 0.21–0.70, *p* = 0.002, respectively) and CVEs (sHR 0.57, 95%CI 0.44–0.73, *p* < 0.001 and sHR 0.44, 95%CI 0.34–0.57, *p* < 0.001, respectively) in both men and women. No difference was found about bleeding events in men and women (sHR 1.18, 95%CI 0.82–1.71, *p* = 0.370 and sHR 1.24, 95%CI 0.822–1.86, *p* = 0.310). Multivariable Cox regression analysis confirmed the inverse association between DOAC and all-cause of death in men and women (HR 0.45, 95%CI 0.34–0.59, *p* < 0.001 and HR 0.25, 95%CI 0.19–0.34, *p* < 0.001) (full models reported in Supplementary Table 4, Panel A). In Fine–Gray multivariable models, DOAC use in men was associated with a non-significant trend toward lower risk of CV death compared to VKA use (sHR 0.56, 95%CI 0.31–1.01, *p* = 0.055). In contrast, among women, DOAC use was significantly associated with a lower risk of CV death (sHR 0.30, 95%CI 0.16–0.56, *p* < 0.001). Regarding CVEs, DOAC use was associated with a significantly lower risk in both men (sHR 0.57, 95%CI 0.44–0.75, *p* < 0.001) and women (sHR 0.34, 95%CI 0.26–0.45, *p* < 0.001). No significant difference in bleeding risk was observed for DOAC versus VKA in either men (sHR 1.21, 95%CI 0.84–1.75, *p* = 0.310) or women (sHR 1.25, 95%CI 0.83–1.88, *p* = 0.290) (Supplementary Table 4, Panel B, C and D).

### Frailty

Among 4244 AF patients, 731 patients had at least one frailty element. In the frailty subgroup, at univariable Cox regression analysis, DOAC use was not associated with a significantly lower risk of all-cause mortality (HR 0.81, 95%CI 0.55–1.19, *p* = 0.289). Similarly, no significant differences were observed between DOAC and VKA use for CV death (sHR 0.49, 95%CI 0.22–1.07, *p* = 0.072), the risk of CVEs (sHR 0.89, 95%CI 0.60–1.32, *p* = 0.570) or bleeding events (sHR 0.62, 95%CI 0.33–1.18, *p* = 0.140) at Fine–Gray univariable analysis.

### Charlson Comorbidity Index (CCI)

In our cohort all patients had a CCI ≥ 3, with a mean CCI of 5.7 ± 1.5 point, without significant difference between DOAC and VKA. At univariable Cox regression analysis, evaluating separately patients with CCI below and above the threshold value of 5, we found similar reduction of all-cause of death in patients treated with DOAC compared to VKA (HR 0.32, 95%CI 0.24–0.44, *p* < 0.001 and HR 0.45, 95%CI 0.35–0.58, *p* < 0.001). At multivariable Cox regression analysis, DOAC use was associated with a lower risk of all-cause of death compared to VKA in both patients with CCI below and above threshold (HR 0.24, 95%CI 0.17–0.34, *p* < 0.001 and HR 0.43, 95%CI 0.33–0.55, *p* < 0.001, respectively) (Supplementary Table 5, Panel A). In multivariable Fine–Gray models accounting for prespecified competing risks (Supplementary Table 5, Panels B, C and D), DOAC use was associated with a significantly lower risk of CV death both in patients below the threshold (sHR 0.28, 95%CI 0.12–0.63, *p* = 0.002) and in those above (sHR 0.57, 95%CI 0.34–0.96, *p* = 0.036). Similarly, DOAC use was associated with a reduced risk of CVEs regardless of comorbidity burden, with stronger effect sizes in patients with lower CCI (sHR 0.31, 95%CI 0.23–0.43, *p* < 0.001) than those with higher (sHR 0.58, 95%CI 0.45–0.74, *p* < 0.001). No significant differences in bleeding risk were observed between DOAC and VKA users in either subgroup (below threshold: sHR 1.39, 95%CI 0.93–2.06, *p* = 0.100; above threshold: sHR 1.21, 95%CI 0.82–1.78, *p* = 0.340).

## Discussion

The results of our contemporary nationwide registry show that the majority of elderly and very elderly patients are now started on DOACs over VKAs. However, some frailty elements influence the choice of oral anticoagulants in this high-risk subgroup of patients. Moreover, the use of DOACs was inversely associated with adverse clinical outcomes, such as mortality and CVEs, with no difference in bleeding risk compared to VKAs. Similar results were obtained in the subgroup of patients aged 90 years or elderly.

The choice of anticoagulation in elderly patients with AF may be challenging, with warfarin being still used for thromboprophylaxis in elderly AF patients[23]. Moreover, in randomized clinical trials, VKAs have a favourable efficacy profile compared to antiplatelet agents regarding the prevention of ischemic stroke (number needed to treat 40–46 compared to aspirin treatment)[24].

A recent study showed an increase in the use of DOAC for elderly patients from 32.4% in 2011 to 43.6% in 2019[16]. Compared to a recent general medicine registry[17] which showed that still < 50% of elderly AF patients were on oral anticoagulants, and that a minority of these patients were treated with DOACs, our registry including patients attending specialized outpatients clinics for the management of antithrombotic therapies shows that the majority of elderly AF patients are currently started on DOACs. This finding highlights that the management of elderly patients by specialized anticoagulation services may increase the rate of DOAC use in frail and old patients[25].

We also found that some frailty elements influence the choice of anticoagulants. Thus, tendency to falls, living alone and the absence of familial/social support were inversely associated with DOACs use.

Thus, from our analysis it seems evident that the risk of falls is still an important factor for clinicians when evaluating anticoagulant therapy. Previous evidence shows that DOACs are still safe and effective in AF patients at risk of falling[26, 27]. A Markov analysis showed that elderly patients on rivaroxaban and apixaban should fall 45 and 458 times, respectively, for the quality-adjusted life-years of a direct oral anticoagulant to be lower than that of aspirin[28].

Conversely, reduction of mobility (i.e. wheelchair users) was directly associated with DOAC use. This factor may reflect a difficulty of patients to attend frequent visits to the outpatients’ clinic for blood sample collection and INR monitoring for the management of warfarin therapy, leading physician to prefer fixed-dose DOAC requiring less laboratory monitoring.

The association between type of anticoagulant and adverse clinical outcomes in elderly and very elderly AF patients is still matter of discussion. A previous study including 4130 patients aged 75 years or elderly showed that treatment with VKAs improved clinical ischemic outcomes compared to untreated ones, without increasing the risk of bleeding[29]. In addition, the use of DOACs in the very elderly population of patients with AF has still areas of uncertainty. A previous nationwide cohort study showed in patients aged ≥ 80 years old a similar risk of ischemic stroke/systemic embolism for patients starting reduced dose of apixaban and dabigatran and a lower risk for those started on rivaroxaban with dabigatran associated with a lower risk of haemorrhagic stroke[30]. However, apixaban and rivaroxaban were associated with an increased risk of death[30].

Another piece of information is given by the association between oral anticoagulants deprescription and increased risk of all-cause mortality in a recent retrospective multicentre cohort study including 1578 elderly patients[31]. These results suggest that deprescribing should be considered only in patients with severe frailty, limited life expectancy and presence of persistent bleeding risk factors, such as cerebral microbleeds[32].

In our study, we found that the use of DOAC inversely associated with mortality risk, both all-cause and CV, and with CVEs, without increasing bleeding risk. This analysis confirmed a previous one performed in 2019 with a smaller sample size and lower number of clinical events[33]. Our results indicate that when properly managed anticoagulation with DOAC is safe also in very elderly patients with AF. Similarly, the subgroup analyses provide additional insights into the consistency of the association between DOAC use and clinical outcomes across clinically relevant strata. Although not always reaching statistical significance, a consistent trend toward reduced considered CV outcomes with DOAC use compared to VKA was observed across subgroups defined by frailty status, sex and comorbidity burden. These findings suggest that the favourable CV profile of DOACs may extend across diverse patient populations, without a clear excess in bleeding risk, though some subgroups may derive greater relative benefit. Potential reasons may explain the favourable effect of DOAC on clinical outcomes. Firstly, an abnormal pharmacokinetics was observed in older patients, characterized by a reduction of drug absorption and bioavailability [34, 35], an increase in fat mass and a decrease in lean body mass and total body water[36] and a decrease of hepatic metabolism of drugs, especially phase I metabolism mediated by cytochrome P450 (CYP) enzymes [34, 37]. In particular, the hepatic impairment, that only in advanced stages changed the DOAC pharmacokinetics, could reduce VKA clearance, while the reduced activity of the vitamin K redox recycling system could increase the sensitivity to VKA and potentially increase the risk of overdosing[38]. In addition, elderly patients were treated with polypharmacy that could increase the risk of drug-drug interactions and compliance reduction[38], especially if treated with VKA, that had a narrow therapeutic range that required frequent dose adjustment and had drug-drug interactions with several drugs.

Our cohort had an acceptable median TiTR, as defined by previous evidence[39], and our study suggested a potential clinical benefit of DOAC also over a well-managed VKA therapy. However, this benefit observed in the DOACs group may be led by both peculiar pharmacokinetics of DOACs and not optimal anticoagulation quality (i.e. TiTR > 70%) in elderly AF patients treated with VKA.

Similar associations were found in patients aged ≥ 90 years. A previous nationwide study performed in Taiwan in nonagenarian patients showed that the use of DOAC compared to warfarin was associated with a lower risk of intracranial haemorrhage and similar risk of ischemic stroke but no data on mortality were reported[40].

We found a favourable NNT for all endpoints at 12 and 24 months, in overall population and in the subgroup nonagenarian AF patients, in whom the benefit was higher, especially for all-cause of death and CVEs. The benefit of DOACs was more evident at 24 months with lower NNT for all endpoints. In a previous meta-analysis[41], the NNT at 2 years ranged from 32 to 16 in an unselected AF population, while in our cohort of elderly AF was 11.0 in those aged ≥ 80 years reaching 4.2 in those aged ≥ 90 years AF patients, showing an ever higher benefit of the use of DOACs in this fragile population.

Our findings have clinical implications. Elderly and very elderly patients with AF should be started on DOACs when AF is detected and there are no contraindications. A comprehensive geriatric assessment is mandatory to find out some frailty elements that may influence the choice of anticoagulation. Due to the study design, patients were enrolled at the time of first oral anticoagulant prescription. The study’s findings demonstrated the choice to prescribe DOACs instead of VKAs, also in very elderly, is not associated with a disutility risk, countering concerns about the effective benefits against the higher costs of the former. Consistently, recent data demonstrated the cost-effectiveness of DOACs vs VKAs in the elderly[42].

### Limitations

Some limitations should be acknowledged. First, we could only analyse some aspects of frailty as factors influencing the choice of oral anticoagulants in this cohort but could not characterize patients due to the lack of other elements potentially representing residual confounders such as mood, nutrition and continence that are part of the geriatric comprehensive evaluation. In particular, in our study we used single frailty elements instead of a comprehensive frailty assessment tool. This non-standardized definition despite provides some important insights on the influence of frailty elements on DOAC use in real-world clinical practice and limits the comparability of our results with other studies and the results obtained from frailty subgroup analysis.

Second, the observational nature of the study precludes definitive conclusions about causality, and the potential influence of unmeasured confounders cannot be entirely ruled out. Third, no data about adherence were collected and the benefit observed may be associated with a higher adherence to anticoagulant treatment. In addition, although we found no clinical benefit of DOAC in patients with at least one frailty elements, this subgroup analysis may be influenced by the small sample size that reduced statistical power and did not allow to consider several potential confounders in the analysis. In addition, the higher competing risks and the low life expectancy in this subgroup could affect the role of DOAC in frailty patients. For this reason, further studies on older patients are required to establish the role of DOAC in this particular cohort of patients.

Finally, because the participating centres primarily serve Italian populations, the cohort lacked ethnic diversity, which may restrict the applicability of our results to other ethnicities.

In conclusion, DOACs are increasingly prescribed to elderly AF patients and are associated with a reduction of adverse clinical outcomes.

## Supplementary Information

Below is the link to the electronic supplementary material.

ESM 1 (DOCX 94.5 KB)

## Data Availability

Data available on reasonable request.

## Appendix

List of participating centres and investigators who contributed to the START2 AF Registry.

Benilde Cosmi^1^, Daniela Poli^2^, Elena Lotti^2^, Martina Berteotti^2^, Rossella Marcucci^2^, Walter Ageno^3^, Giovanna Colombo^3^, Doris Barcellona^4^, Giovanni Barillari^5^, Alessandra Poz^5^, Salvatore Bradamante⁶, Eugenio Bucherini⁷, Monica Vastola⁷, Luca Bucherini⁷, Paola Casasco⁸, Antonio Ciampa⁹, Antonio Chistolini^1^⁰, Alessandra Serrao^1^⁰, Luciano Crippa^11^, Raimondo De Cristofaro^12^, Erica De Candia^12^, Valeria De Micheli^13^, Igor Diemberger^14^, Giuseppe Boriani^14^, Marcello Di Nisio^15^, Ettore Porreca^15^, Anna Falanga^1^⁶, Teresa Lerede^1^⁶, Elvira Grandone^1^⁷, Donatella Colaizzo^1^⁷, Antonio Insana^1^⁸, Nicola Lucio Liberato^1^⁹, Domenico Lione^2^⁰, Rosa Maria Lombardi^21^, Giacomo Lucarelli^22^, Giuseppe Malcangi^23^, Catello Mangione^24^, Giuliana Martini^25^, Marco Marzolo^2^⁶, Giovanni Nante^2^⁷, Vincenzo Oriana^2^⁸, Carmelo Paparo^2^⁹, Paolo Pedico^3^⁰, Simona Pedrini^31^, Vittorio Pengo^32^, Antonietta Piana^33^, Francesco Cibecchini^33^, Simona Pezzella^34^, Pasquale Pignatelli^35^, Daniele Pastori^35^, Vincenza Rossi^3^⁶, Lucia Ruocco^3^⁷, Paolo Chiarugi^3^⁷, Serena Rupoli^3^⁸, Domizio Serra^3^⁹, Carmine Spataro^4^⁰, Margherita Reduzzi^4^⁰, Chiara Ambaglio^4^⁰, Sophie Testa^41^, Oriana Paoletti^41^, Andrea Toma^42^, Vincenzo Toschi^43^, Rita Carlotta Santoro^44^, Claudio Vasselli^45^, Maddalena Loredana Zighetti^4^⁶, Marco Cattaneo^4^⁶, Gianmarco Podda^4^⁶.

1. Division of Angiology and Blood Coagulation, IRCCS Azienda Ospedaliero-Universitaria S. Orsola-Malpighi, Bologna, Italy
2. SOD Malattie Aterotrombotiche, Azienda Ospedaliero Universitaria-Careggi, Firenze, Italy
3. SSD Degenza Breve Internistica, Dipartimento di Medicina Interna, Ospedale di Circolo e Fondazione Macchi, ASST Sette Laghi, Varese, Italy
4. UOC Medicina Interna, Centro Emocoagulopatie, AOU Cagliari, Cagliari
5. Ambulatorio Malattie Emorragiche e Trombotiche, Medicina Trasfusionale di Udine, Presidio Ospedaliero Universitario “Santa Maria della Misericordia”, Udine, Italy
6. Centro Trasfusionale, Centro Emostasi e Trombosi, Ospedale SS Annunziata, Taranto, Italy
7. SS Medicina Vascolare e Angiologia, AUSL Romagna—Ravenna, Italy
8. Centro Diagnosi Trombosi e Sorveglianza Terapia Anticoagulanti, Immunoematologia Trasfusionale, Ospedale SS Antonio e Margherita, Tortona (AL)
9. Dipartimento Medicina di Laboratorio, Laboratorio Analisi, Ospedale San Giuseppe Moscati, Avellino, Italy
10. Dipartimento di Medicina Traslazionale e di Precisione Sapienza Università di Roma, Roma, Italy
11. Ambulatorio Emostasi e Trombosi, UO Patologie Trombotiche Ospedale San Raffaele, Milano, Italy
12. UOSD Malattie Emorragiche e Trombotiche, Dipartimento Diagnostica per Immagini, Radioterapia Oncologica ed Ematologia, Fondazione Policlinico Universitario Agostino Gemelli IRCCS, Roma, Italy
13. Centro Emostasi e Trombosi, Medicina Generale, ASST Lecco, Lecco, Italy
14. Dipartimento Cardiotoraco Vascolare, IRCCS Azienda Ospedaliero-Universitaria S. Orsola-Malpighi, Bologna, Italy
15. Dipartimento di Medicina e scienze dell’invecchiamento, SSD Medicina Interna University “G.D’Annunzio” di Chieti-Pescara, Pescara, Italy
16. Università Bicocca Milano, Dept. Medicine and Surgery, Monza and Divisione di Immunoematologia e Medicina Trasfusionale & Centro Emostasi e Trombosi, ASST Papa Giovanni XXIII, Bergamo, Italy
17. Centro Trombosi, Casa del Sollievo e della Sofferenza, S.Giovanni Rotondo (Foggia), Italy
18. SC Laboratorio Analisi Chimico-Cliniche e Microbiologia, A.O. Ordine Mauriziano, Torino, Italy
19. UO Medicina Interna, Ambulatorio Terapia Anticoagulante Orale, AO Provincia di Pavia, Pavia, Italy
20. UOC di Patologia Clinica, Ospedale A Perrino, Brindisi, Italy
21. Centro Emostasi e Trombosi, UO Medicina Interna, Azienda Ospedaliero-Universitaria di Parma, Italy
22. Centro Trombosi, Ospedale Regionale F. Miulli, Acquaviva delle Fonti (Bari), Italy
23. Centro Emofilia e Trombosi, Policlinico di Bari, Bari, Italy
24. Trasfusionale, Ospedale di Galatina, Galatina (LE), Italy
25. Centro Emostasi, Spedali Civili Di Brescia, Brescia, Italy
26. UOS Angiologia Medica, Ospedale di Rovigo, Italy
27. Dipartimento di Medicina, Azienda Ospedaliero-Universitaria di Padova, Padova, Italy
28. Centro Emofilia—Presidio Ospedaliero Morelli, Reggio Calabria
29. Laboratorio Analisi, Ospedale Maggiore Chieri, Torino, Italy
30. UO di Medicina Trasfusionale, Presidio Ospedaliero Dimiccoli, Barletta (Bari), Italy
31. Servizio di Laboratorio, Istituto Ospedaliero Fondazione Poliambulanza, Brescia, Italy
32. Dipartimento di Scienze Cardio-Toraco-Vascolari, AOU Padova, Padova, Italy
33. Centro Prevenzione e Cura Malattie Tromboemboliche e Centro TAO, I.R.C.C.S. Azienda Ospedaliera Universitaria San Martino-IST, Genova, Italy
34. UO Medicina Trasfusionale, Centro TAO, PO Battipaglia, Battipaglia, (Salerno), Italy
35. Centro Trombosi, Dipartimento SCIAC, Sapienza Università di Roma, Italy
36. Centro Emostasi e Trombosi, UOC Ematologia, Azienda Ospedaliera di Cosenza, Cosenza, Italy
37. UO di Analisi Chimico Cliniche, Azienda Ospedaliero Universitaria Pisana, Pisa, Italy
38. Clinica di Ematologia, AOU Ospedali Riuniti di Ancona, Ancona, Italy
39. Servizio Analisi, Ospedale Evangelico Internazionale, Sede di Castelletto, Genova, Italy
40. SIMT ASST Bergamo Ovest—Ospedale di Treviglio, Pavia Italy
41. Centro Emostasi e Trombosi, UUOO Laboratorio Analisi chimico-cliniche e microbiologiche, ASST Cremona, Cremona, Italy
42. UOC di Patologia Clinica, Ambulatorio Terapia Anticoagulante Orale, O.C. “L. Cazzavillan” Arzignano, (Vicenza), Italy
43. Dipartimento di Medicina Trasfusionale e di Ematologia, ASST Santi Paolo e Carlo, Milano, Italy
44. Centro Emostasi e Trombosi, UO Emofilia e Patologie della Coagulazione, Dipartimento di Ematologia, Oncologia e Medicina Trasfusionale, Azienda Ospedaliera “Pugliese Ciaccio”, Catanzaro, Italy
45. Laboratorio Patologia Clinica, Policlinico Casilino—Roma, Roma Italy
46. U.O. Medicina II, Azienda Ospedaliera San Paolo di Milano, ASST Santi Paolo e Carlo, Milano, Italy
