# Supplementary material for: Frailty elements, direct oral anticoagulants and mortality risk in atrial fibrillation patients aged ≥ 80 or ≥ 90 years from the nationwide Italian START registry: a propensity score matching analysis
Source: GeroScience. 2025 Oct 22;48(4):5407–25. doi: 10.1007/s11357-025-01936-8 (PMC13574756; doi:10.1007/s11357-025-01936-8)
Supplement: Supplementary file 1 [file 11357_2025_1936_MOESM1_ESM.docx]

**Frailty elements, direct oral anticoagulants, and mortality risk in atrial fibrillation patients aged ≥80 or ≥90 years from the nationwide Italian START registry: a propensity score matching analysis.**

**Correspondence to:**

Prof. Daniele Pastori, MD, PhD, FESC.

Department of Clinical Internal, Anesthesiological, and Cardiovascular Sciences, Sapienza University of Rome, Rome, Italy.

Email: [daniele.pastori@uniroma1.it](mailto:daniele.pastori@uniroma1.it)

## Supplementary Table 1. Baseline characteristics of unmatched and propensity score-matched cohorts.

|  | All Patients | | | Matched Patients | | |
| --- | --- | --- | --- | --- | --- | --- |
|  | **VKA,  N = 1,528** | **DOAC,  N = 2,716** | **SMD** | **VKA,  N = 1,496** | **DOAC,  N = 1,496** | **SMD** |
| Age (years) | 84.1 ± 3.2 | 85.1 ± 4.0 | 0.4767 | 84.2 ± 3.2 | 84.5 ± 3.7 | 0.0642 |
| Women | 845 (55%) | 1,486 (55%) | 0.2347 | 835 (56%) | 796 (53%) | -0.0524 |
| Paroxysmal AF | 407 (27%) | 893 (33%) | -0.0118 | 402 (27%) | 482 (32%) | 0.1138 |
| Previous CAD | 316 (21%) | 403 (15%) | 0.1329 | 298 (20%) | 325 (22%) | 0.0508 |
| Heart failure | 439 (29%) | 662 (24%) | -0.1644 | 422 (28%) | 480 (32%) | 0.0903 |
| Peripheral artery disease | 119 (7.8%) | 145 (5.3%) | -0.1015 | 110 (7.4%) | 113 (7.6%) | 0.0089 |
| CKD* | 301 (20%) | 699 (26%) | -0.1089 | 298 (20%) | 332 (22%) | 0.0520 |
| Alcohol use | 107 (7.0%) | 94 (3.5%) | 0.1381 | 88 (5.9%) | 88 (5.9%) | 0.0000 |
| Dementia | 64 (4.2%) | 177 (6.5%) | -0.1938 | 64 (4.3%) | 75 (5.0%) | 0.0298 |
| Wheelchair users | 54 (3.5%) | 153 (5.6%) | 0.0943 | 54 (3.6%) | 65 (4.3%) | 0.0319 |
| Living alone | 117 (7.7%) | 143 (5.3%) | 0.0910 | 112 (7.5%) | 119 (8.0%) | 0.0210 |
| Social/familial support | 1,058 (69%) | 2,001 (74%) | -0.1071 | 1,039 (69%) | 993 (66%) | -0.0698 |

*AF: atrial fibrillation, CAD: Coronary Artery Disease, CKD: chronic kidney disease, DOAC: direct oral anticoagulants, SMD: Standardised Mean Difference, VKA: vitamin K antagonist.*

**defined as estimated glomerular filtration rate (eGFR)<60 ml/min*

## Supplementary Table 2. Univariable analyses: Cox regression for all-cause mortality (Panel A) and Fine–Gray models for cardiovascular death (Panel B), cardiovascular events (Panel C), and bleeding (Panel D), comparing DOACs vs VKAs.

| **Panel A** | **Hazard Ratio** | **95% Confidence Interval**  **Low High** | | **p-value** |
| --- | --- | --- | --- | --- |
| Apixaban  Rivaroxaban  Edoxaban  Dabigatran | 0.41  0.46  0.31  0.38 | 0.31  0.34  0.20  0.27 | 0.54  0.62  0.48  0.56 | <0.001  <0.001  <0.001  <0.001 |
| **Panel B – CV Death** | **sHR** |  |  |  |
| Apixaban | 0.39 | 0.21 | 0.73 | 0.003 |
| Rivaroxaban | 0.72 | 0.41 | 1.25 | 0.240 |
| Edoxaban | 0.12 | 0.03 | 0.52 | 0.004 |
| Dabigatran | 0.62 | 0.33 | 1.19 | 0.150 |
| **Panel C - CVEs** | **sHR** |  |  |  |
| Apixaban | 0.52 | 0.40 | 0.66 | <0.001 |
| Rivaroxaban | 0.60 | 0.46 | 0.78 | <0.001 |
| Edoxaban | 0.37 | 0.24 | 0.55 | <0.001 |
| Dabigatran | 0.47 | 0.34 | 0.65 | <0.001 |
| **Panel D – Bleeding events** | **sHR** |  |  |  |
| Apixaban | 0.97 | 0.67 | 1.42 | 0.890 |
| Rivaroxaban | 1.04 | 0.68 | 1.58 | 0.870 |
| Edoxaban | 1.56 | 1.00 | 2.43 | 0.051 |
| Dabigatran | 1.50 | 1.01 | 2.24 | 0.047 |

*CV: Cardiovascular, CVE: Cardiovascular Event, DOACs: direct oral anticoagulants, sHR, subdistribution Hazard Ratio; VKA: vitamin K antagonists*

## Supplementary Table 3. Univariable analyses: Cox regression for all-cause mortality (Panel A) and Fine–Gray models for cardiovascular death (Panel B), cardiovascular events (Panel C), and bleeding (Panel D), comparing factor Xa inhibitors vs dabigatran.

| **Panel A** | **Hazard Ratio** | **95% Confidence Interval**  **Low High** | | **p-value** |
| --- | --- | --- | --- | --- |
| Apixaban  Rivaroxaban  Edoxaban  Dabigatran | 1.04  1.18  0.76  Ref | 0.68  0.75  0.44  Ref | 1.61  1.84  1.33  Ref | 0.846  0.474  0.341  - |
| **Panel B – CV Death** |  |  |  |  |
| Apixaban | 0.63 | 0.28 | 1.44 | 0.280 |
| Rivaroxaban | 1.16 | 0.54 | 2.49 | 0.710 |
| Edoxaban | 0.21 | 0.05 | 0.99 | 0.048 |
| Dabigatran | Ref | Ref | Ref | - |
| **Panel C - CVEs** |  |  |  |  |
| Apixaban  Rivaroxaban  Edoxaban  Dabigatran | 1.08  1.26  0.75  Ref | 0.74 0.85  0.45  Ref | 1.59  1.88  1.25  Ref | 0.694  0.248  0.271  - |
| **Panel D – Bleeding events** |  |  |  |  |
| Apixaban | 0.65 | 0.41 | 1.04 | 0.073 |
| Rivaroxaban | 0.70 | 0.42 | 1.15 | 0.150 |
| Edoxaban | 1.05 | 0.63 | 1.77 | 0.840 |
| Dabigatran | Ref | Ref | Ref | - |

*CV: Cardiovascular, CVE: Cardiovascular Event, sHR, subdistribution Hazard Ratio.*

## Supplementary Table 4. Multivariable analyses: stepwise Cox regression for all-cause mortality (Panel A) and Fine–Gray models for cardiovascular death (Panel B), cardiovascular events (Panel C), and bleeding (Panel D), according to sex.

| **Panel A** | **HR** | **95% Confidence Interval**  **Low High** | | **p-value** |
| --- | --- | --- | --- | --- |
| Men | | | | |
| *DOAC (vs VKA)* | 0.45 | 0.34 | 0.59 | <0.001 |
| *Age* | 1.10 | 1.07 | 1.14 | <0.001 |
| *Previous CAD* | 1.77 | 1.31 | 2.38 | <0.001 |
| *Paroxysmal AF* | 0.70 | 0.51 | 0.96 | 0.027 |
| *Previous stroke/TIA* | 1.64 | 1.21 | 2.22 | 0.001 |
| *PAD* | 1.65 | 1.10 | 2.47 | 0.015 |
| *Anemia* | 1.47 | 1.13 | 1.91 | 0.004 |
| *CKD** | 0.62 | 0.45 | 0.86 | 0.005 |
| *Wheelchair use* | 1.85 | 1.09 | 3.14 | 0.023 |
| *COPD/OSAS* | 2.17 | 1.62 | 2.89 | <0.001 |
| *LLT* | 0.62 | 0.45 | 0.84 | 0.002 |
| Women | | | | |
| *DOAC* | 0.25 | 0.19 | 0.34 | <0.001 |
| *Age* | 1.13 | 1.09 | 1.17 | <0.001 |
| *Diabetes mellitus* | 1.39 | 1.01 | 1.91 | 0.043 |
| *Dementia* | 1.86 | 1.21 | 2.85 | 0.005 |
| *Wheelchair use* | 2.13 | 1.39 | 3.25 | <0.001 |
| *COPD/OSAS* | 1.72 | 1.21 | 2.45 | 0.003 |
| *LLT* | 0.67 | 0.49 | 0.91 | 0.011 |
| **Panel B** | **sHR** | **95% Confidence Interval**  **Low High** | | **p-value** |
| Men | | | | |
| *DOAC (vs VKA)* | 0.56 | 0.31 | 1.01 | 0.055 |
| *Age* | 1.11 | 1.03 | 1.19 | 0.006 |
| *Previous CAD* | 1.82 | 1.08 | 3.05 | 0.024 |
| *Paroxysmal AF* | 0.65 | 0.34 | 1.26 | 0.200 |
| *Previous stroke/TIA* | 1.63 | 0.93 | 2.83 | 0.086 |
| *PAD* | 1.87 | 0.94 | 3.73 | 0.075 |
| *Anemia* | 1.91 | 1.12 | 3.24 | 0.017 |
| *CKD** | 0.52 | 0.25 | 1.09 | 0.082 |
| *Wheelchair use* | 2.39 | 1.00 | 5.71 | 0.049 |
| *COPD/OSAS* | 1.53 | 0.85 | 2.76 | 0.160 |
| *LLT* | 0.70 | 0.41 | 1.20 | 0.200 |
| Women | | | | |
| *DOAC* | 0.30 | 0.16 | 0.56 | <0.001 |
| *Age* | 1.11 | 1.05 | 1.18 | <0.001 |
| *Diabetes mellitus* | 0.71 | 0.30 | 1.69 | 0.430 |
| *Dementia* | 1.88 | 0.80 | 4.44 | 0.150 |
| *Wheelchair use* | 2.15 | 0.91 | 5.08 | 0.081 |
| *COPD/OSAS* | 1.09 | 0.46 | 2.55 | 0.850 |
| *LLT* | 0.42 | 0.18 | 0.98 | 0.044 |
| **Panel C** | **sHR** | **95% Confidence Interval**  **Low High** | | p-value |
| Men | | | | |
| *DOAC (vs VKA)* | 0.57 | 0.44 | 0.75 | <0.001 |
| *Age* | 1.09 | 1.05 | 1.12 | <0.001 |
| *Previous CAD* | 1.73 | 1.30 | 2.29 | <0.001 |
| *Paroxysmal AF* | 0.81 | 0.61 | 1.08 | 0.1500 |
| *Previous stroke/TIA* | 1.54 | 1.16 | 2.05 | 0.003 |
| *PAD* | 1.79 | 1.25 | 2.59 | 0.002 |
| *Anemia* | 1.43 | 1.11 | 1.83 | 0.005 |
| *CKD** | 0.58 | 0.43 | 0.77 | <0.001 |
| *Wheelchair use* | 1.70 | 1.02 | 2.84 | 0.043 |
| *COPD/OSAS* | 1.97 | 1.48 | 2.62 | <0.001 |
| *LLT* | 0.64 | 0.48 | 0.85 | 0.003 |
| Women | | | | |
| *DOAC* | 0.34 | 0.26 | 0.45 | <0.001 |
| *Age* | 1.12 | 1.09 | 1.16 | <0.001 |
| *Diabetes mellitus* | 1.23 | 0.90 | 1.67 | 0.190 |
| *Dementia* | 1.71 | 1.12 | 2.61 | 0.012 |
| *Wheelchair use* | 2.18 | 1.49 | 3.19 | <0.001 |
| *COPD/OSAS* | 1.63 | 1.17 | 2.28 | 0.004 |
| *LLT* | 0.69 | 0.52 | 0.92 | 0.012 |
| **Panel D** | **sHR** | **95% Confidence Interval**  **Low High** | | p-value |
| Men | | | | |
| *DOAC (vs VKA)* | 1.21 | 0.84 | 1.75 | 0.310 |
| *Age* | 1.02 | 0.97 | 1.07 | 0.480 |
| *Previous CAD* | 1.14 | 0.75 | 1.75 | 0.540 |
| *Paroxysmal AF* | 1.67 | 1.16 | 2.41 | 0.006 |
| *Previous stroke/TIA* | 1.16 | 0.74 | 1.80 | 0.520 |
| *PAD* | 1.42 | 0.82 | 2.46 | 0.210 |
| *Anemia* | 2.18 | 1.51 | 3.15 | <0.001 |
| *CKD** | 0.81 | 0.53 | 1.23 | 0.310 |
| *Wheelchair use* | 1.10 | 0.49 | 2.49 | 0.820 |
| *COPD/OSAS* | 1.17 | 0.75 | 1.84 | 0.480 |
| *LLT* | 0.92 | 0.62 | 1.37 | 0.680 |
| Women | | | | |
| *DOAC* | 1.25 | 0.83 | 1.88 | 0.290 |
| *Age* | 1.02 | 0.97 | 1.07 | 0.400 |
| *Diabetes mellitus* | 1.29 | 0.79 | 2.12 | 0.310 |
| *Dementia* | 0.90 | 0.36 | 2.29 | 0.830 |
| *Wheelchair use* | 0.33 | 0.08 | 1.35 | 0.120 |
| *COPD/OSAS* | 0.80 | 0.39 | 1.67 | 0.560 |
| *LLT* | 1.16 | 0.74 | 1.81 | 0.520 |

*AF: Atrial fibrillation, CKD: Chronic kidney disease, COPD/OSAS: chronic obstructive pulmonary disease/obstructive sleep apnoea syndrome, CV: Cardiovascular, CVE: Cardiovascular Event, DOAC: direct oral anticoagulants, HR: Hazard Ratio, LLT: lipid lowering therapy, PAD: peripheral artery disease, sHR, subdistribution Hazard Ratio, TIA: transient ischemic attack, VKA: vitamin K antagonist.*

**defined as estimated glomerular filtration rate (eGFR)<60 ml/min*

## Supplementary Table 5. Multivariable analyses: stepwise Cox regression for all-cause mortality (Panel A) and Fine–Gray models for cardiovascular death (Panel B), cardiovascular events (Panel C), and bleeding (Panel D), according to Charlson Comorbidity Index (CCI).

| **Panel A** | **HR** | **95% Confidence Interval**  **Low High** | | **p-value** |
| --- | --- | --- | --- | --- |
| CCI ≤ 5 | | | | |
| *DOAC (vs VKA)* | 0.24 | 0.17 | 0.34 | <0.001 |
| *Age* | 1.14 | 1.09 | 1.18 | <0.001 |
| *Diabetes* | 1.58 | 1.01 | 2.48 | 0.046 |
| *Previous CAD* | 1.80 | 1.08 | 2.97 | 0.023 |
| *Anaemia* | 1.32 | 1.00 | 1.76 | 0.053 |
| *Cancer* | 0.57 | 0.36 | 0.90 | 0.017 |
| *COPD/OSAS* | 2.07 | 1.26 | 3.38 | 0.004 |
| *Dementia* | 3.68 | 1.70 | 7.98 | 0.001 |
| *Tendency to falls* | 2.00 | 1.03 | 3.88 | 0.040 |
| *Wheelchair use* | 2.28 | 1.07 | 4.83 | 0.032 |
| *LLT* | 0.58 | 0.40 | 0.84 | 0.004 |
| *RAASi* | 0.74 | 0.56 | 0.97 | 0.031 |
| *Digoxin* | 1.48 | 1.00 | 2.19 | 0.049 |
| CCI > 5 | | | | |
| *DOAC (vs VKA)* | 0.43 | 0.33 | 0.55 | <0.001 |
| *Female sex* | 0.60 | 0.46 | 0.78 | <0.001 |
| *Age* | 1.11 | 1.07 | 1.14 | <0.001 |
| *Anemia* | 1.30 | 1.01 | 1.66 | 0.040 |
| *Cancer* | 1.34 | 1.02 | 1.77 | 0.038 |
| *COPD/OSAS* | 1.54 | 1.18 | 2.01 | 0.001 |
| *CKD** | 0.48 | 0.35 | 0.64 | <0.001 |
| *Wheelchair use* | 1.83 | 1.27 | 2.64 | 0.001 |
| **Panel B** | **sHR** | **95% Confidence Interval**  **Low High** | | **p-value** |
| CCI ≤ 5 | | | | |
| *DOAC (vs VKA)* | 0.28 | 0.12 | 0.63 | 0.002 |
| *Age* | 1.13 | 1.05 | 1.22 | 0.002 |
| *Diabetes* | 1.02 | 0.37 | 2.78 | 0.970 |
| *Previous CAD* | 1.09 | 0.32 | 3.75 | 0.890 |
| *Anaemia* | 1.43 | 0.76 | 2.70 | 0.270 |
| *Cancer* | 0.42 | 0.13 | 1.34 | 0.140 |
| *COPD/OSAS* | 1.29 | 0.40 | 4.11 | 0.670 |
| *Dementia* | 3.84 | 0.88 | 16.8 | 0.073 |
| *Tendency to falls* | 0.70 | 0.10 | 4.94 | 0.720 |
| *Wheelchair use* | 1.15 | 0.15 | 8.91 | 0.900 |
| *LLT* | 0.60 | 0.27 | 1.34 | 0.210 |
| *RAASi* | 1.07 | 0.59 | 1.91 | 0.830 |
| *Digoxin* | 1.72 | 0.81 | 3.67 | 0.160 |
| CCI > 5 | | | | |
| *DOAC (vs VKA)* | 0.57 | 0.34 | 0.96 | 0.036 |
| *Female sex* | 0.53 | 0.31 | 0.91 | 0.021 |
| *Age* | 1.10 | 1.03 | 1.17 | 0.003 |
| *Anemia* | 1.16 | 0.71 | 1.89 | 0.560 |
| *Cancer* | 1.38 | 0.78 | 2.43 | 0.270 |
| *COPD/OSAS* | 1.03 | 0.60 | 1.79 | 0.910 |
| *CKD** | 0.34 | 0.17 | 0.68 | 0.002 |
| *Wheelchair use* | 2.17 | 1.08 | 4.37 | 0.031 |
| **Panel C** | **sHR** | **95% Confidence Interval**  **Low High** | | **p-value** |
| CCI ≤ 5 |  |  |  |  |
| *DOAC (vs VKA)* | 0.31 | 0.23 | 0.43 | <0.001 |
| *Age* | 1.13 | 1.09 | 1.16 | <0.001 |
| *Diabetes* | 1.58 | 1.05 | 2.37 | 0.028 |
| *Previous CAD* | 1.61 | 0.99 | 2.62 | 0.054 |
| *Anaemia* | 1.26 | 0.97 | 1.65 | 0.087 |
| *Cancer* | 0.58 | 0.36 | 0.92 | 0.022 |
| *COPD/OSAS* | 1.95 | 1.20 | 3.15 | 0.007 |
| *Dementia* | 3.02 | 1.39 | 6.54 | 0.005 |
| *Tendency to falls* | 1.64 | 0.86 | 3.12 | 0.130 |
| *Wheelchair use* | 2.87 | 1.52 | 5.40 | 0.001 |
| *LLT* | 0.62 | 0.45 | 0.87 | 0.005 |
| *RAASi* | 0.71 | 0.55 | 0.92 | 0.009 |
| *Digoxin* | 1.41 | 0.97 | 2.05 | 0.069 |
| CCI > 5 | | | | |
| *DOAC (vs VKA)* | 0.58 | 0.45 | 0.74 | <0.001 |
| *Female sex* | 0.58 | 0.45 | 0.74 | <0.001 |
| *Age* | 1.09 | 1.06 | 1.12 | <0.001 |
| *Anemia* | 1.25 | 0.99 | 1.58 | 0.063 |
| *Cancer* | 1.35 | 1.04 | 1.74 | 0.022 |
| *COPD/OSAS* | 1.45 | 1.13 | 1.86 | 0.004 |
| *CKD** | 0.42 | 0.32 | 0.56 | <0.001 |
| *Wheelchair use* | 1.67 | 1.20 | 2.34 | 0.003 |
| **Panel D** | **sHR** | **95% Confidence Interval**  **Low High** | | **p-value** |
| CCI ≤ 5 | | | | |
| *DOAC (vs VKA)* | 1.39 | 0.93 | 2.06 | 0.100 |
| *Age* | 1.02 | 0.98 | 1.07 | 0.330 |
| *Diabetes* | 1.10 | 0.57 | 2.12 | 0.780 |
| *Previous CAD* | 0.82 | 0.35 | 1.93 | 0.650 |
| *Anaemia* | 1.50 | 1.01 | 2.23 | 0.047 |
| *Cancer* | 1.14 | 0.68 | 1.91 | 0.630 |
| *COPD/OSAS* | 1.07 | 0.46 | 2.49 | 0.870 |
| *Dementia* | 1.55 | 0.47 | 5.12 | 0.470 |
| *Tendency to falls* | 0.33 | 0.05 | 2.46 | 0.280 |
| *Wheelchair use* | 0.46 | 0.07 | 3.20 | 0.440 |
| *LLT* | 0.72 | 0.44 | 1.19 | 0.200 |
| *RAASi* | 0.80 | 0.54 | 1.18 | 0.270 |
| *Digoxin* | 1.16 | 0.62 | 2.17 | 0.650 |
| CCI > 5 | | | | |
| *DOAC (vs VKA)* | 1.21 | 0.82 | 1.78 | 0.340 |
| *Female sex* | 0.53 | 0.36 | 0.79 | 0.002 |
| *Age* | 1.00 | 0.95 | 1.06 | 0.950 |
| *Anemia* | 1.40 | 0.97 | 2.03 | 0.076 |
| *Cancer* | 1.02 | 0.65 | 1.60 | 0.920 |
| *COPD/OSAS* | 0.91 | 0.58 | 1.43 | 0.690 |
| *CKD** | 0.63 | 0.42 | 0.95 | 0.027 |
| *Wheelchair use* | 0.73 | 0.34 | 1.59 | 0.430 |

*AF: Atrial fibrillation, CAD: coronary artery disease, CKD: Chronic kidney disease, COPD/OSAS: chronic obstructive pulmonary disease/obstructive sleep apnoea syndrome, CV: Cardiovascular, CVE: Cardiovascular Event, DOAC: direct oral anticoagulants, HR: Hazard Ratio, LLT: lipid lowering therapy, PAD: peripheral artery disease, RAASi: renin-angiotensin-aldosterone system inhibitors, sHR, subdistribution Hazard Ratio, TIA: transient ischemic attack, VKA: vitamin K antagonist.*

**defined as estimated glomerular filtration rate (eGFR)<60 ml/min*

## Supplementary Figure 1. Love Plot: covariate balance before and after matching comparing patients treated with VKAs and DOACs.

## Supplementary Figure 2. Kaplan–Meier curves by type of anticoagulant for all-cause mortality (Panel A), cardiovascular death (Panel B), cardiovascular events (Panel C), and any bleeding (Panel D) in patients aged ≥90 years.

**A**

p<0.001

**B**

p<0.001

**C**

p<0.001

**D**

p=0.535
